# Protection against Ultraviolet A-Induced Skin Apoptosis and Carcinogenesis through the Oxidative Stress Reduction Effects of *N*-(4-bromophenethyl) Caffeamide, a Propolis Derivative

**DOI:** 10.3390/antiox9040335

**Published:** 2020-04-20

**Authors:** Yueh-Hsiung Kuo, Hung-Lung Chiang, Po-Yuan Wu, Yin Chu, Qiao-Xin Chang, Kuo-Ching Wen, Chien-Yih Lin, Hsiu-Mei Chiang

**Affiliations:** 1 Department of Chinese Pharmaceutical Sciences and Chinese Medicine Resources, China Medical University, Taichung 40402, Taiwan; kuoyh@mail.cmu.edu.tw; 2 Department of Biotechnology, Asia University, Taichung 41354, Taiwan; 3 Chinese Medicine Research Center, China Medical University, Taichung 40447, Taiwan; 4 Department and Graduate School of Safety Health and Environmental Engineering, National Yulin University of Science and Technology, Yulin 64002, Taiwan; hlchiang@yuntech.edu.tw; 5 Department of Dermatology, China Medical University Hospital, Taichung 40402, Taiwan; wu.poyuan@gmail.com; 6 School of Medicine, China Medical University, Taichung 40402, Taiwan; 7 Department of Cosmeceutics, China Medical University, Taichung 40402, Taiwan; dooo517@outlook.com (Y.C.); nancystar597@gmail.com (Q.-X.C.); kcwen0520@mail.cmu.edu.tw (K.-C.W.); yihlin@asia.edu.tw (C.-Y.L.); 8 Ph.D Program for Biotechnology Industry, China Medical University, Taichung 40402, Taiwan

**Keywords:** *N*-(4-bromophenethyl) caffeamide, oxidative stress, DNA damage, apoptosis, inflammation

## Abstract

Ultraviolet A (UVA) is a major factor in skin aging and damage. Antioxidative materials may ameliorate this UV damage. This study investigated the protective properties of *N*-(4-bromophenethyl) caffeamide (K36H) against UVA-induced skin inflammation, apoptosis and genotoxicity in keratinocytes. The protein expression or biofactor concentration related to UVA-induced skin damage were identified using an enzyme-linked immunosorbent assay and western blotting. K36H reduced UVA-induced intracellular reactive oxygen species generation and increased nuclear factor erythroid 2–related factor 2 translocation into the nucleus to upregulate the expression of heme oxygenase-1, an intrinsic antioxidant enzyme. K36H inhibited UVA-induced activation of extracellular-signal-regulated kinases and c-Jun N-terminal kinases, reduced the overexpression of matrix metalloproteinase (MMP)-1 and MMP-2 and elevated the expression of the metalloproteinase-1 tissue inhibitor. Moreover, K36H inhibited the phosphorylation of c-Jun and downregulated *c*-Fos expression. K36H attenuated UVA-induced Bax and caspase-3 expression and upregulated antiapoptotic protein B-cell lymphoma 2 expression. K36H reduced UVA-induced DNA damage. K36H also downregulated inducible nitric oxide synthase, cyclooxygenase-2 and interleukin-6 expression as well as the subsequent generation of prostaglandin E_2_ and nitric oxide. We observed that K36H ameliorated UVA-induced oxidative stress, inflammation, apoptosis and antiphotocarcinogenic activity. K36H can potentially be used for the development of antiphotodamage and antiphotocarcinogenic products.

## 1. Introduction

The sun emits various light wavebands, of which ultraviolet (UV) irradiation is the most hazardous for the skin [1]. Long wavelength ultraviolet light is linked to oxidative stress and damage to the skin, including premature skin aging, DNA damage, mutations and even skin cancer [2,3]. UVA indirectly induces DNA damage. The damage is caused by reactive oxygen species (ROS) generated following the interaction of cellular molecules with UVA [4]. Cellular molecules, such as porphyrins and flavins, absorb UVA and produce ROS through Jablonski reactions [4]. ROS generation causes several oxidative damage-related disorders of cells and the skin. Therefore, identifying potential countermeasures against UVA-radiation-mediated oxidative damage to skin cells is essential. The oxidation process may trigger intrinsic apoptosis of cells by influencing the membrane potential of mitochondria to release cytochrome C and then activate downstream proapoptotic caspases [5,6].

Apoptosis is a programmed process for maintaining organismal homeostasis and eliminating damaged cells. Many proteins are involved in UVA-induced apoptosis, including caspases, Bax and B-cell lymphoma-2 (Bcl-2) [7]. Bax is a death factor that forms a heterodimer with Bcl-2, a survival factor. After proapoptotic signaling, Bax and Bcl-2 families undergo modification to gain their full apoptotic function [8]. Bcl-2 expression is regulated by complex factors, including ROS and UV light exposure. UVA irradiation can induce dermal cell apoptosis through Bcl-2 downregulation and Bax upregulation to modulate the expression and genes of caspases [9].

Self-oxidative stress defense systems counterbalance reactive oxidants in the body and are triggered when skin cells encounter enormous oxidative stress [10,11]. Nuclear factor erythroid 2-related factor 2 (Nrf2) is a transcription factor that promotes Nrf2 ubiquitination when combined with Kelch-like ECH-associated protein 1 (Keap1) [12,13]. As skin cells experience oxidative stress, Keap1 conformation changes and it separates from Nrf2, which subsequently translocates into the nucleus in the cells and binds to an antioxidant response element (ARE). Phase II detoxifying enzymes such as heme oxygenase-1 (HO-1) subsequently produce [14,15]. UVA induced a high level of HO-1 protein and exhibited anti-inflammatory and antiapoptotic properties through the capture of excessive free radicals [16]. In addition, HO-1 can protect murine skin cells from tumor formation triggered by oxidative stress and also protect human skin cells from UVA irradiation damage [17]. Moreover, HO-1 inhibits apoptosis in mouse fibroblasts induced by tumor necrosis factor-α [18].

Excess ROS generation upregulates nuclear factor kappa B (NF-κB), which leads to tumor necrosis factor-α and interleukin (IL)-6 production and further induces prostaglandin [19] and cyclooxygenase-2 (COX-2) production, causing inflammation [20]. Moreover, UVA radiation upregulates the mRNA of inducible nitric oxide synthase (iNOS) and then induces nitric oxide (NO) overproduction, which can lead to cellular apoptosis and inflammation [21,22,23]. UV irradiation activates mitogen-activated protein (MAP) kinases and then triggers downstream protein expression, such as that of activator protein-1 (AP-1; formed by Fos and Jun family proteins) and matrix metalloproteinases (MMPs), to degrade the extracellular matrix, causing skin damage [24,25].

**Figure 1:**
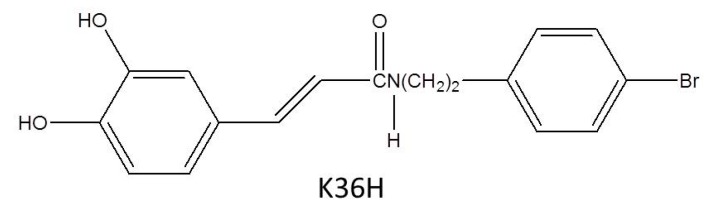
Structure of caffeamide (K36H).

Caffeamide exhibits potent antioxidative activity and scavenging of free radicals [26]. N-(4-bromophenethyl) caffeamide (K36H) (Figure 1) is a caffeamide derivative that inhibits the breakdown of type I procollagen and stimulates the synthesis of collagen in human skin fibroblasts after exposure to ultraviolet B (UVB) [27]. K36H also exhibits anti-inflammation in human skin fibroblasts. Furthermore, it inhibits melanogenesis and melanogenesis-related proteins such as tyrosinase and TRP-1 in B16F0 cells [17]. UVA is the most prevalent form of solar radiation that reaches the earth surface; therefore, protection against UVA-induced skin damage is essential [28,29]. Thus, this study was intended to discover the reparative effects of K36H on apoptosis and DNA damage in human epidermal keratinocytes caused by UVA-induced oxidation.

## 2. Materials and Methods

### 2.1. Materials

The synthesis process and identification of K36H was illustrated in a report [30]. K36H was dissolved in dimethyl sulfoxide (DMSO) for the experiments; the final concentration of DMSO was less than 0.1%. Reagents, serum and mediums for cell culture were supplied by Gibco, Invitrogen (Carlsbad, CA, USA). DMSO, trypan blue solution, 2′,7′-dichlorofluorescin diacetate (DCFDA), dithiothreitol, phenylmethylsulfonyl fluoride, paraformaldehyde and leupeptin were obtained from Sigma Chemical Co. (St. Louis, MO, USA). Igepal CA-630, tris, sodium dodecyl sulfate (SDS), thiazolyl blue tetrazolium bromides (MTT) and Tween20 were supplied by USB Corporation (Cleveland, OH, USA). A PageRuler prestained protein ladder and WesternBright enhanced chemiluminescence (ECL) blotting detection kit were supplied by Amersham Biosciences (Little Chalfont, Buckinghamshire, UK). Bradford reagent for measurement of the protein concentration was purchased from Bio-Rad Laboratories (Hercules, CA, USA). All other chemicals used in this study were of reagent grade.

### 2.2. Cell Culture and Viability Assay

HaCaT cell line, an immortal human epidermal keratinocyte purchased from Cell Lines Service (Eppelheim, Germany), was cultured in Dulbecco’s modified Eagle’s medium (DMEM) with 10% fetal bovine serum and 100 U/mL penicillin–streptomycin at 37 °C in a humidified atmosphere containing 5% CO_2_/95% air. The cells were cultured in 100-mm dishes and 24-well plates for attachment. For measurement of the survival rate of cells, an MTT assay was applied [31,32]. Various concentrations of K36H were incubated with HaCaT cells for 24 h. MTT solution was added to the plate, which was then converted to insoluble formazan crystals by cells. The formazan that formed was dissolved in 10% SDS-HCl, and the optical density was then measured at 570 nm with a microplate meter (Tecan, Grödig, Austria).

### 2.3. UVA Exposure

The medium was removed, and phosphate-buffered saline (PBS) was added to wash the cells. The cells were exposed to UVA irradiation by using UV Crosslinkers XLE-1000A, in which the major wavelength of UVA lamps is 365 nm (Spectroline, Westbury, NY, USA) [31]. The cells were treated with UVA for approximately 45 min (10 J/cm^2^) at a distance of 15.2 cm and were subsequently cultured in serum-free DMEM containing various concentrations of K36H for the indicated time.

### 2.4. Measurement of Intracellular ROS Generation

To survey the ability of K36H to eliminate UVA-induced intracellular oxidative stress, the cells were cultured in 24-well plates and irradiated with UVA irradiation [31]. DMEM containing 10-μM DCFDA was added into the plates after incubation with various concentrations of K36H for 3 h. The intensity of fluorescence was excited at 488 nm and emitted at 520 nm wavelengths (Thermo Electron Corporation, Vantaa, Finland).

### 2.5. Immunofluorescence Staining

HaCaT keratinocytes were grown on a cover slip and treated with 5–50-μM K36H after UVA irradiation. After 24 h, cells were fixed with 4% paraformaldehyde after being washed with PBS. They were then blocked with MPBS (containing 5% nonfat milk solution and 0.3% Triton X-100) for 30 min. PBS was used to wash the cover slip, and incubation was performed with a primary antibody for 30 min and then with a secondary antibody, antirabbit immunoglobulin (IgG) (Alexa Fluor 488, Invitrogen, Carlsbad, CA, USA). Thereafter, the cover slips were washed with PBS to remove the unbound secondary antibody. The cover slips were counterstained using ProLong Gold antifade reagent with 4′,6-diamidino-2-phenylindole and fluorescence was observed with a confocal laser scanning microscope (Leica DMIL, Wetzlar, Germany).

### 2.6. Western Blotting Analysis

After the indicated treatments, the adherent cells were scraped from the dishes and lysed with radioimmunoprecipitation assay buffer on ice. The protein concentration was determined using Bradford assay reagents. The protein from these cells was electrophoresed on SDS-polyacrylamide gels; subsequently, the proteins were transferred to polyvinylidene difluoride membranes and incubated with primary and secondary antibodies. The proteins were determined with the ECL western blotting detection system (LAS-4000, Fujifilm, Japan). The results were analyzed using software (MultiGauge V2.2, Fuji Pharm, Tokyo, Japan).

### 2.7. Comet Assay

The comet assay was applied to detect the DNA strand breaks in HaCaT cells according to the manufacturer’s protocol with minor modifications as previous described (Trevigen, Gaithersburg, MD, USA) [33]. Cells were combined with agarose and then pipetted to a three-well slide. The slides were stored in the dark at 4 °C for 15 min. Later, they were immersed in lysis solution for 45 min at 4 °C. After removal from the lysis buffer, alkaline solution was added, and the slide was placed in the dark at 4 °C for 30 min. Subsequently, the slides were placed in the electrophoresis slide tray, and alkaline electrophoresis solution (at a temperature of 4 °C) was added. The voltage of the power supply was set to 14 V and applied for 25 min. Slides were immersed twice in water and then in 75% ethanol for 5 min. Vista DNA green buffer was added, and slides were viewed using an epifluorescence microscope.

### 2.8. NO Measurement

The NO content of the cultured medium was detected after treatment with UVA and various K36H concentrations according to the manual protocol from the Greiss reagent supplier (Promega, Madison, Wisconsin, USA) as previously described [31]. N-(1-Naphthyl) ethylenediamine solution and sulfanilamide were added to the cell culture medium and mixed. The absorbance was detected using an enzyme-linked immunosorbent assay (ELISA) reader with a filter at 540 nm.

### 2.9. Prostaglandin E_2_ Measurement

The prostaglandin E_2_ (PGE_2_) content was measured per the manual protocol from the manufacturer as previously described (Cayman, Ann Arbor, MI, USA) [31]. A 96-well plate coated with goat polyclonal antimouse IgG secondary antibody and the cell culture medium was added. PGE_2_ primary antibody and acetylcholinesterase tracer were added into the plate and then maintained at 4 °C for 18 h. Thereafter, the absorbance was determined at 420 nm by using an ELISA reader after Ellman’s reagent was added.

### 2.10. Data and Statistical Methods

The values of the results were expressed as the mean ± standard deviation of independent experiments performed at least three times. The statistical analysis was performed using one-way analysis of variance and the Tukey post hoc test (*p* < 0.05).

## 3. Results and Discussion

### 3.1. Effect of K36H Treatment on Cytotoxicity of Keratinocytes

**Figure 2:**
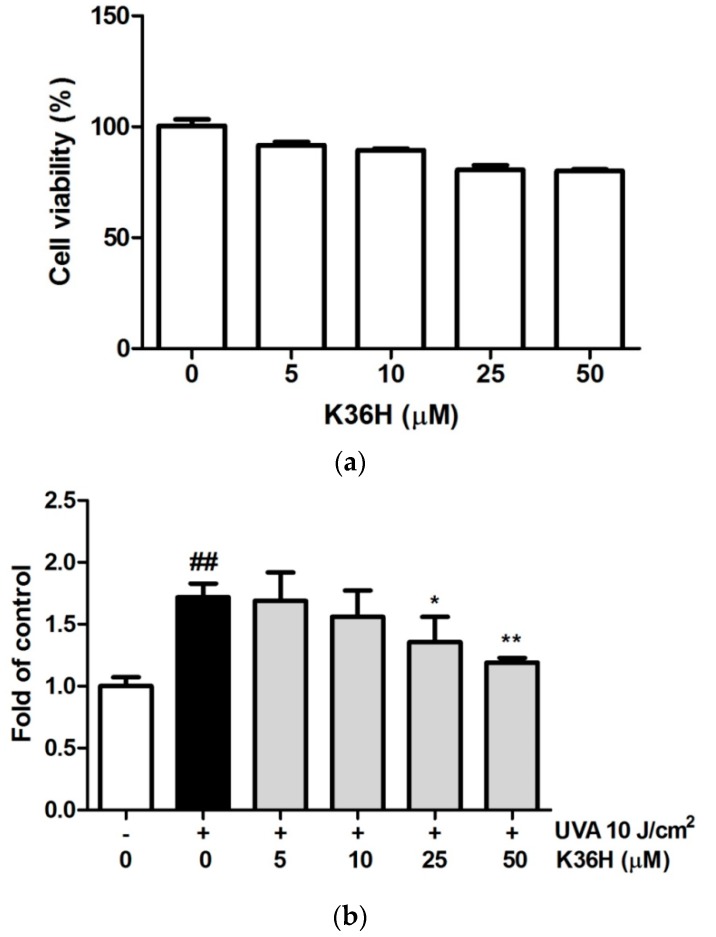
(**a**) Cell survival rate (%) of K36H in HaCaT cells. (**b**) K36H reduced intracellular oxidative stress induced by ultraviolet A (UVA)-irradiation in human epidermal keratinocytes. (## *p* < 0.01 compared with the nonirradiated group. * *p* < 0.05; ** *p* < 0.01 compared with the nontreatment group.).

After treatment with various concentrations of K36H, the cell survival rate was assayed with the MTT test. The viability of cells treated with 0-, 5-, 10-, 25- and 50-μM K36H was 100.0 ± 0.9, 91.6 ± 1.6, 89.4 ± 0.8, 80.5 ± 2.2 and 80.2 ± 1.1, respectively. Survival rates higher than 80% indicated that K36H did not exhibit cytotoxicity (Figure 2a). In another study, K36H exhibited no cytotoxicity in Hs68 cells [34].

### 3.2. Reduction of UVA-Induced Intracellular ROS Generation with K36H Treatment

The DCFDA assay was applied to determine ROS generation in keratinocytes. In this study, the keratinocytes were exposed to UVA irradiation (10 J/cm^2^) and treated with K36H. The ROS production was detected using the DCFDA assay. Figure 2b shows that the ROS levels induced by UVA-irradiation keratinocytes increased by 1.72-fold. After treatment with 25- and 50-μM K36H, the ROS level significantly decreased to 1.36 and 1.19 times that of the control group. K36H is a derivative from the constituents of propolis. In another study we conducted, K36H exhibited DPPH scavenging and inhibited intracellular ROS generation, which may slow skin aging [34]. Catechol, the functional group of K36H, may provide hydrogen atoms that contribute to free radical scavenging and provide inherent antioxidant potential [35]. This may contribute to the protective activity of K36H from photoaging. In this study, K36H reduced UVA-induced ROS generation in keratinocytes.

UVA harms lipids, DNA and proteins in the skin through the generation of numerous ROS, which is a hallmark of oxidative damage [36]. The generation of ROS and free radicals may cause cytotoxicity and apoptosis in skin cells. In addition, excessive ROS can trigger aging and related disorders, DNA damage, mutation and even tumors. Many studies have shown that substances capable of reversing oxidative stress have potential antiaging and anticancer properties. Topical application of propolis extract was reported to protect mouse skin from lipid peroxidation induced by UV light (290–400 nm) and inflammation [37].

### 3.3. Regulation of Nrf2 and HO-1 Expression and of Nrf-2 Translocation2 with K36H Treatment

**Figure 3:**
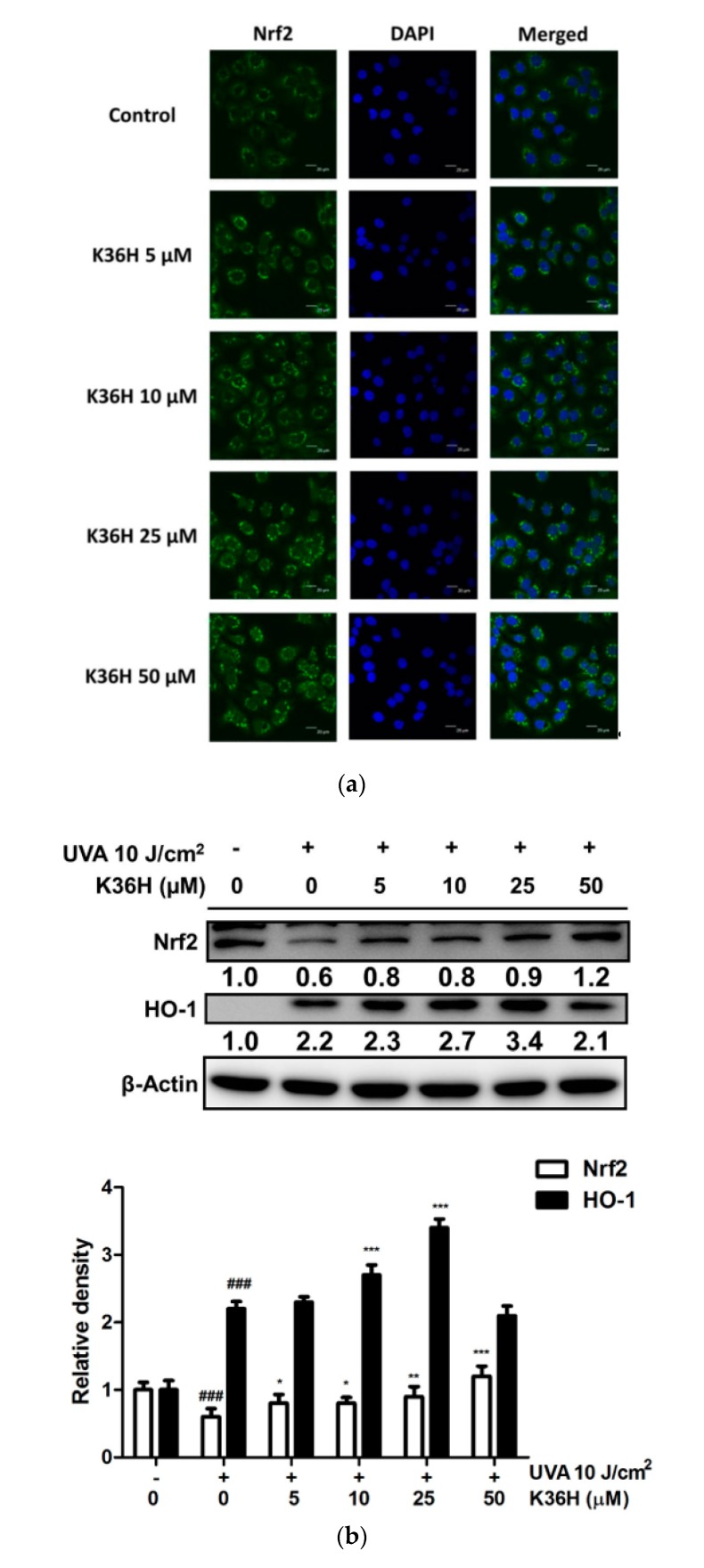
Effect of K36H on (**a**) Nrf2 translocation and (**b**) UVA-upregulated expression of Nrf2 and HO-1 in human epidermal keratinocytes. Significant difference versus the nonirradiated group: ### *p* < 0.001. (* *p* < 0.05; ** *p* < 0.01; *** *p* < 0.001 compared with the nontreatment group).

To investigate the role of the oxidative stress defense system on the antioxidant property of K36H, the translocation and protein expression of Nrf2 and HO-1 were detected. Immunofluorescence staining showed that K36H promoted cellular Nrf2 translocation in keratinocytes (Figure 3a). In addition, UVA reduced Nrf2 expression. However, K36H can inhibit this effect (Figure 3b). For downstream protein expression, we found that HO-1 expression increased to 2.2-fold after 10 J/cm^2^ UVA irradiation and to 2.3-, 2.7- and 3.4-fold after K36H treatment of the control group (Figure 3b). Thus, K36H may ameliorate oxidative stress in keratinocytes through induction of Nrf2 translocation followed by upregulated HO-1 expression.

The expressions of some proteins of antioxidant defense system have been found to be affected by exposure to oxidizing agents. Among the cellular self-defense systems, HO-1 is one of the most pivotal antioxidative proteins. HO-1 is regulated by Nrf2 and antioxidant response element. Nrf2 modulates the transcription of several antioxidant genes protecting cells from oxidative stress [38]. Nrf2 was reported to protect cells from UV irradiation-induced oxidative damage and dysfunction [39]; furthermore, it plays a major role as a stimulant of antiapoptotic proteins from the Bcl-2 family and responds to proinflammatory factors [40]. UVA-induced oxidative damage results in apoptotic cell death. Because K36H is a potent antioxidant, it could prevent UV radiation-induced oxidative damage. In one study, propolis upregulated HO-1 expression in UV-irradiated mouse skin and ameliorated skin damage [37]. Our study showed that K36H effectively upregulates the protein expression of HO-1 in HaCaT cells and induces Nrf2 translocation from cytoplasm into the nucleus. Therefore, K36H protected keratinocytes from UVA-induced oxidative damage through facilitation of Nrf2 translocation and elevation of downstream HO-1 expression.

### 3.4. Antiphotodamage Properties of K36H

#### 3.4.1. Downregulation of MMP Expression with K36H Treatment

**Figure 4:**
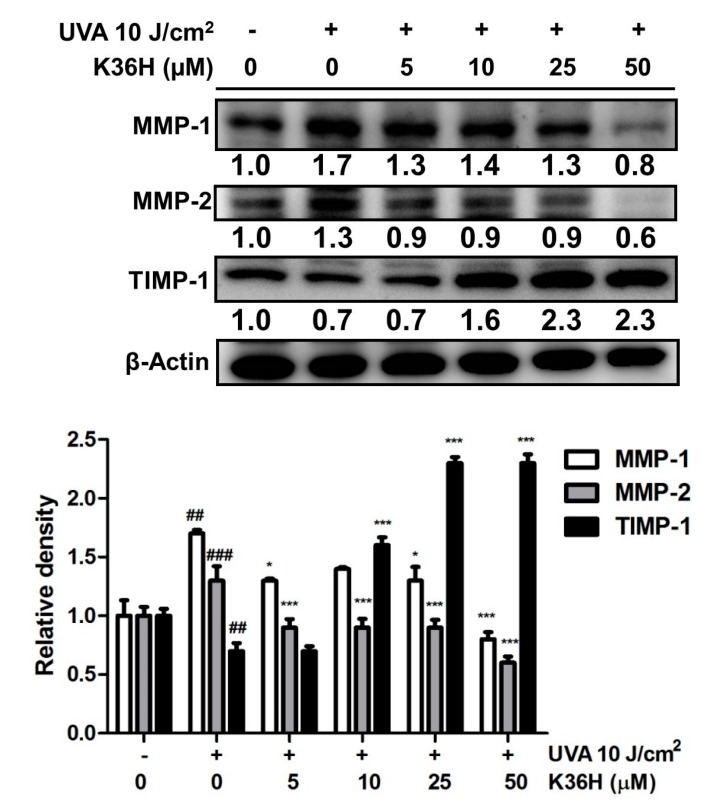
Effect of K36H on UVA-induced matrix metalloproteinase (MMP)-1, MMP-2 and TIMP-1 expression in human epidermal keratinocytes. Significant difference versus the nonirradiated group: ## *p* < 0.01; ### *p* < 0.001. Significant difference versus the nontreatment group: * *p* < 0.05; *** *p* < 0.001.

MMPs are zinc-dependent endogenous proteases related to cell differentiation, proliferation and migration as well as extracellular matrix (ECM) degradation and modification [41]. MMP-1 is the main proteinase that degrades type I and III collagen in the dermis, whereas MMP-2 degrades type IV collagen and gelatin [42]. After irradiation with 10 J/cm^2^ of UVA, the protein expressions of MMP-1 and MMP-2, respectively, increased to 1.7 and 1.3 times those in the control group and decreased to 1.3 and 0.9 time the control group level with 5-μM K36H treatment (Figure 4). Among endogenous MMP inhibitors, the expression of tissue inhibitor of metalloproteinase (TIMP)-1 decreased to 0.7 times the control group levels after UVA exposure and recovered to 1.6 times the control group level after 10-μM K36H treatment.

#### 3.4.2. Reduction of c-Jun and c-Fos Activation with K36H Treatment

**Figure 5:**
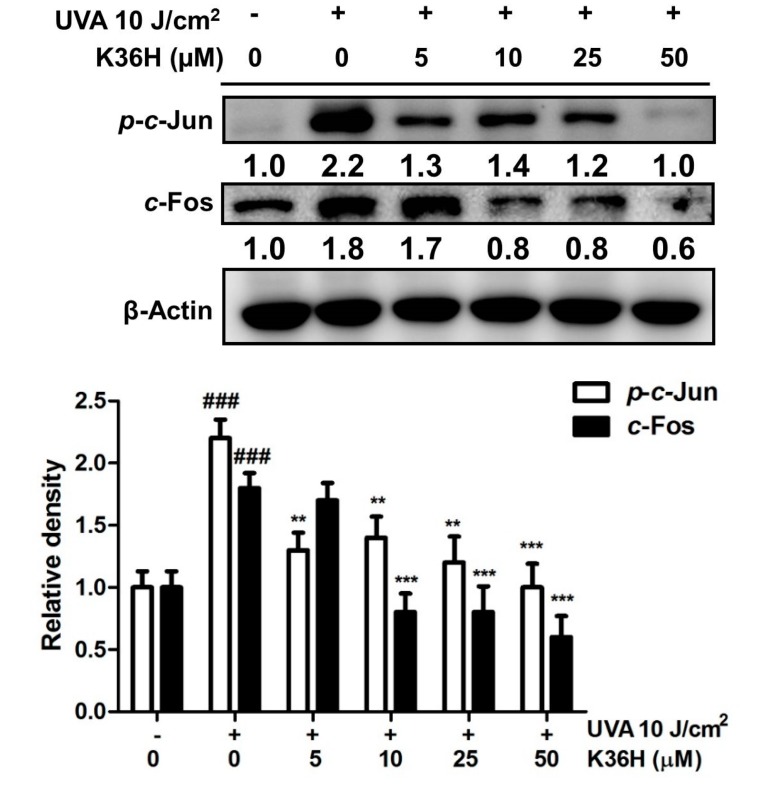
Effect of K36H on UVA-induced p-c-Jun and c-Fos expression in human epidermal keratinocytes. Significant difference versus the nonirradiated group: ### *p* < 0.001. Significant difference versus the nontreatment group: ** *p* < 0.01; *** *p* < 0.001.

As shown in Figure 5, the expression of c-Jun and c-Fos protein increased 2.2- and 1.8-fold after UVA radiation and significantly decreased to 1.4- and 0.8-fold after 10-μM K36H treatment. AP-1 comprises proteins belonging to the c-Fos and c-Jun family. AP-1 translocates from the cytoplasm into the nucleus and modulates downstream genes and the expression of proteins such as MMP-1 and MMP-2, which cause ECM degradation, resulting in skin aging [43,44].

#### 3.4.3. Inhibition of MAP Kinase Phosphorylation with K36H Treatment

**Figure 6:**
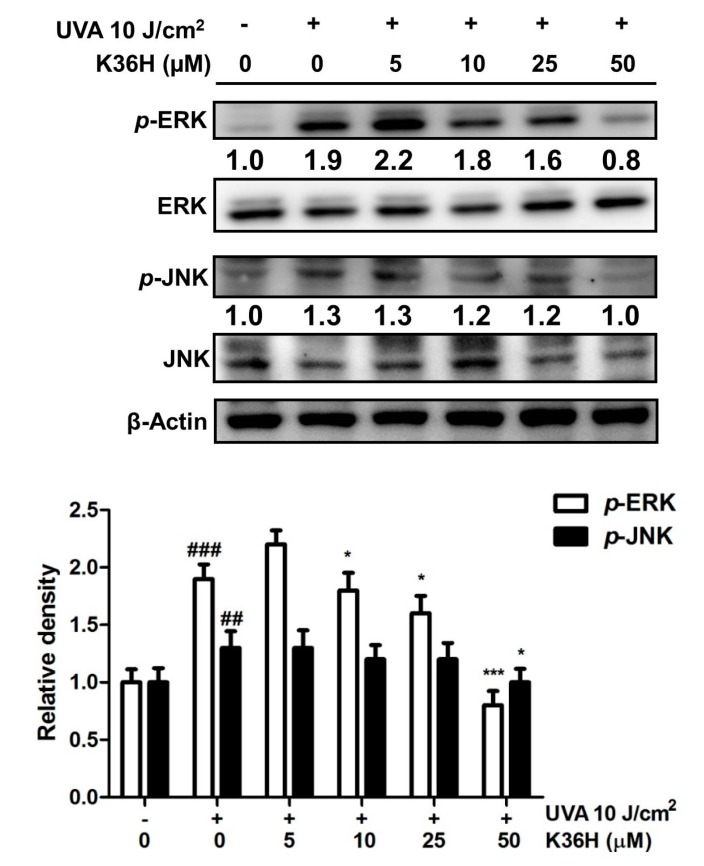
Effect of K36H on UVA-induced phosphorylation of *p*-extracellular-signal-regulated kinases (*p*-ERKs) and *p*-Jun N-terminal kinase (*p*-JNK) expression in human epidermal keratinocytes. Significant difference versus the nonirradiated group: ## *p* < 0.01; ### *p* < 0.001. Significant difference versus the nontreatment group: * *p* < 0.05; *** *p* < 0.001.

The protein expression of *p*-extracellular-signal-regulated kinases (*p*-ERKs) increased to 1.9-fold compared with the control levels after UVA exposure, and it decreased to 0.8-fold of the control levels after 50-μM K36H treatment (Figure 6). The *p*-Jun N-terminal kinase (*p*-JNK) expression increased to 1.3-fold compared with the control levels after UVA light exposure and decreased to 1.0-fold of the control levels after treatment with 50-μM K36H. After UV irradiation, some biofactors such as cytokines and growth factor receptors are activated, resulting in MAP kinase activation and upregulated protein expression. UVA can induce skin aging through the upregulation of MAP kinases and MMP expression in fibroblasts; however, treatment with ginseng protein downregulated MAP kinases and MMP expression to protect skin from UVA-induced photodamage [45]. The results of this study indicated that K36H suppressed UVA-induced MMP-1 and MMP-2 overexpression and restored TIMP-1 protein expression in UVA-exposed HaCaT cells. Another study revealed that K36H restored total collagen in UVB-exposed Hs68 fibroblasts and reduced collagen degradation [34]. This study proved the protective effect of K36H against long-wavelength UV irradiation-induced skin damage through the inhibition of MAP kinases and the AP-1 pathway.

### 3.5. UVA-Induced DNA Damage Inhibition with K36H Treatment

**Figure 7:**
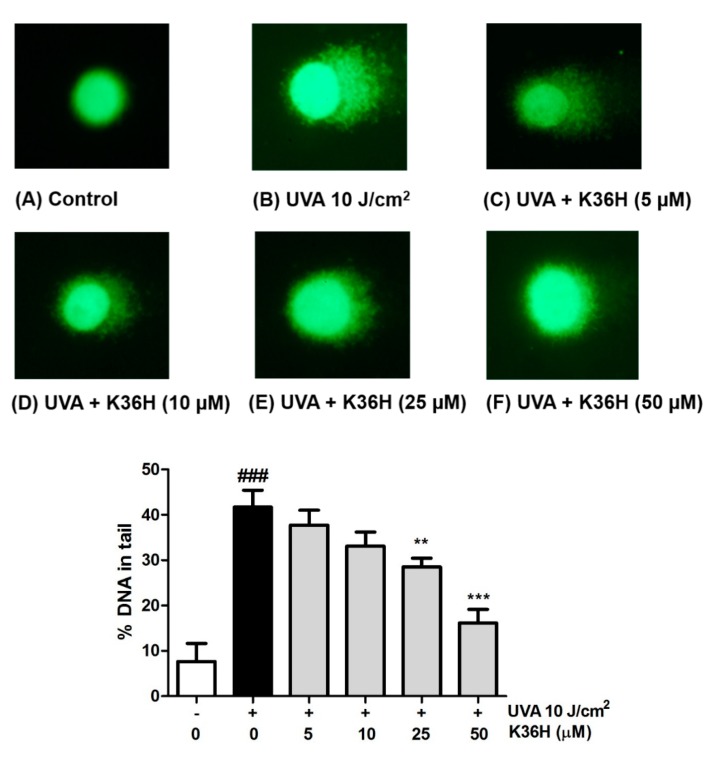
Effect of K36H on UVA-induced cellular DNA damage in human epidermal keratinocytes. Significant difference versus the nonirradiated group: ### *p* < 0.001. Significant difference versus the nontreatment group: ** *p* < 0.01; *** *p* < 0.001.

The protective effects of K36H on DNA damage were assayed using the comet assay. As shown in Figure 7, after UVA irradiation, the DNA in the tail increased to 41.7%; after treatment with 5–50-μM K36H, the DNA in the tail significantly decreased to 37.7%, 33.1%, 28.5% and 16.1%. K36H reduced DNA damage induced by UVA light exposure in keratinocytes.

### 3.6. K36H Regulation of Apoptosis

**Figure 8:**
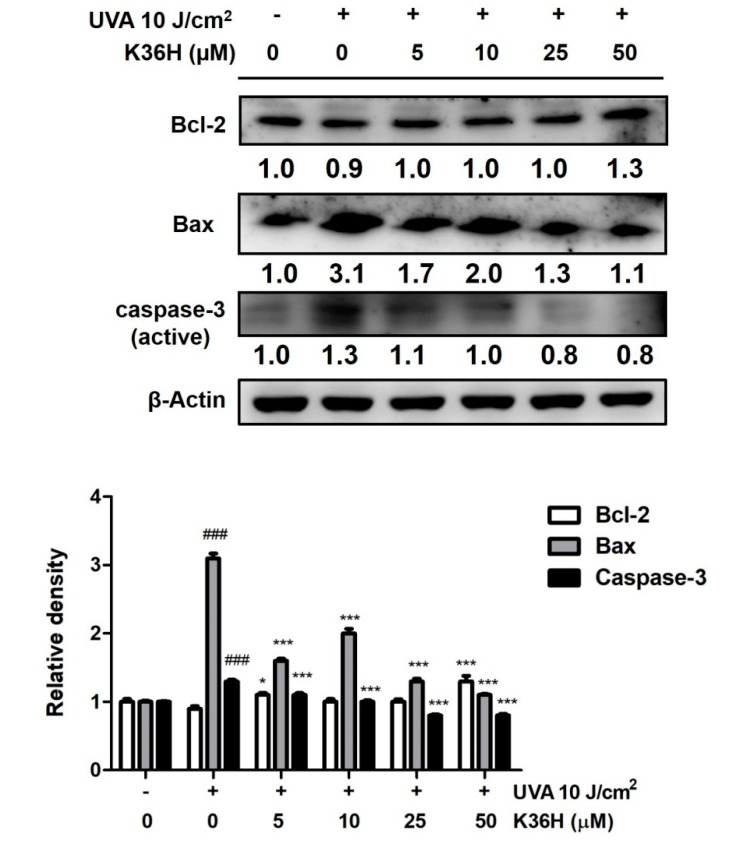
Effect of K36H on UVA-induced Bax, caspase-3 and reduced Bcl-2 protein expression in human epidermal keratinocytes. Significant difference versus the nonirradiated group: ### *p* < 0.001. Significant difference versus the nontreatment group: * *p* < 0.05, *** *p* < 0.001.

**Figure 9:**
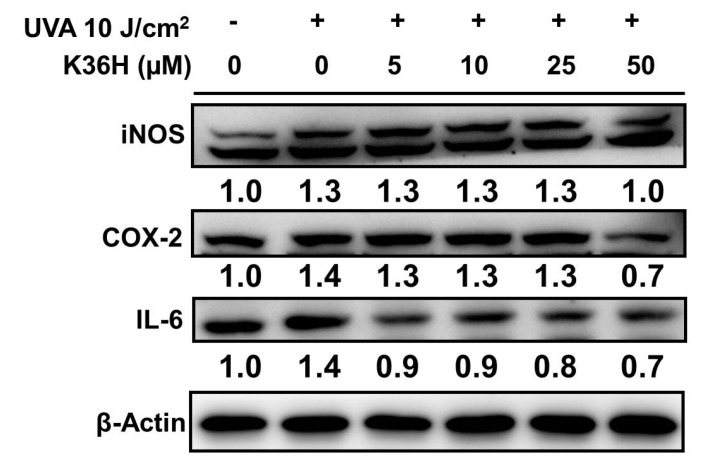

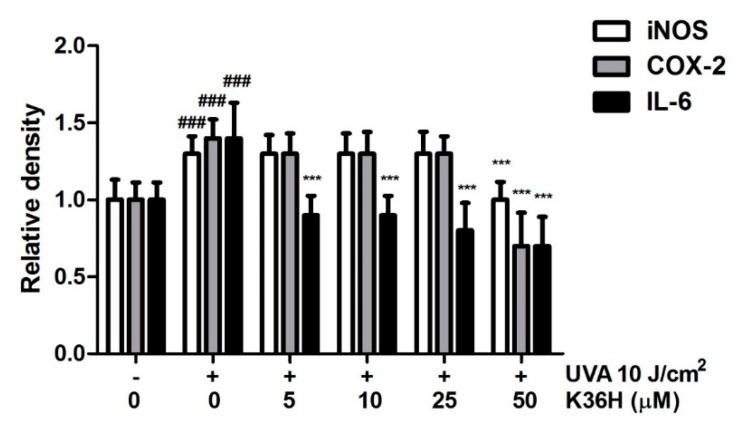
Effect of K36H on UVA-induced iNOS, COX-2 and IL-6 expression in human epidermal keratinocytes. Significant difference versus the nonirradiated group: ### *p* < 0.001. Significant difference versus nontreatment group: *** *p* < 0.001.

Overproduction of proinflammatory cytokines and chemokines has been reported to lead to aging, apoptosis and even cell death. During exposure of cells to UV light, the membrane potential of mitochondria changes, causing cytochrome C to be released into the cytosol and subsequent activate Bax and other apoptotic proteins [46]. In our results, Bax expression increased to 3.1-fold that of the control levels after UVA irradiation and K36H significantly reduced the expression at a dose higher than 5 μM (Figure 8). In addition, caspase-3 expression increased, and K36H treatment significantly reduced the expression at a dose higher than 5 μM. Bcl-2 was downregulated to 0.9-fold of the control levels after 10 J/cm^2^ UVA exposure; K36H treatment restored Bcl-2 expression (Figure 9). After 50 -M K36H treatment, the Bcl-2 expression was upregulated by 1.3-fold compared with the control levels. Thus, K36H may protect skin cells from UVA-induced apoptosis.

UV exposure induced skin cancer through oxidation and inflammatory reactions [2,47]. UV-induced apoptosis involves the contribution of DNA damage, cell surface death receptors and ROS generation [48]. In our results, UVA exposure elevated caspase-3 and Bax expression, inducing apoptosis in keratinocytes, whereas K36H inhibited these effects. UVA exposure enhanced dermal cell apoptosis and the mechanism involved the downregulation of Bcl-2; our results are consistent with other reports in relevant literature [7,48]. K36H treatment can reverse UVA-induced apoptosis through the suppression of Bcl-2 expression.

### 3.7. UVA-induced Inflammation Inhibition by K36H

#### 3.7.1. Reduction of UVA-Induced-Inflammation-Related Protein Expression with K36H Treatment

To analyze the anti-inflammatory effect of K36H, we used western blotting to observe the protein expression of inflammatory mediators, including COX-2, iNOS and IL-6 proteins. The iNOS protein expression increased to 1.3-fold in the keratinocytes of the control group and decreased to 1.0-fold of the control group levels after 50-μM K36H treatment (Figure 9). COX-2 expression was increased 1.4-fold after UVA radiation; it decreased significantly, to 0.7 times that of the control level after 50-μM K36H treatment. The IL-6 expression increased to 1.4-fold after irradiation with 10 J/cm^2^ UVA and was downregulated to 0.9-fold with 5-μM K36H treatment (Figure 9).

#### 3.7.2. Reduction of UVA-Induced NO Production with K36H Treatment

**Figure 10:**
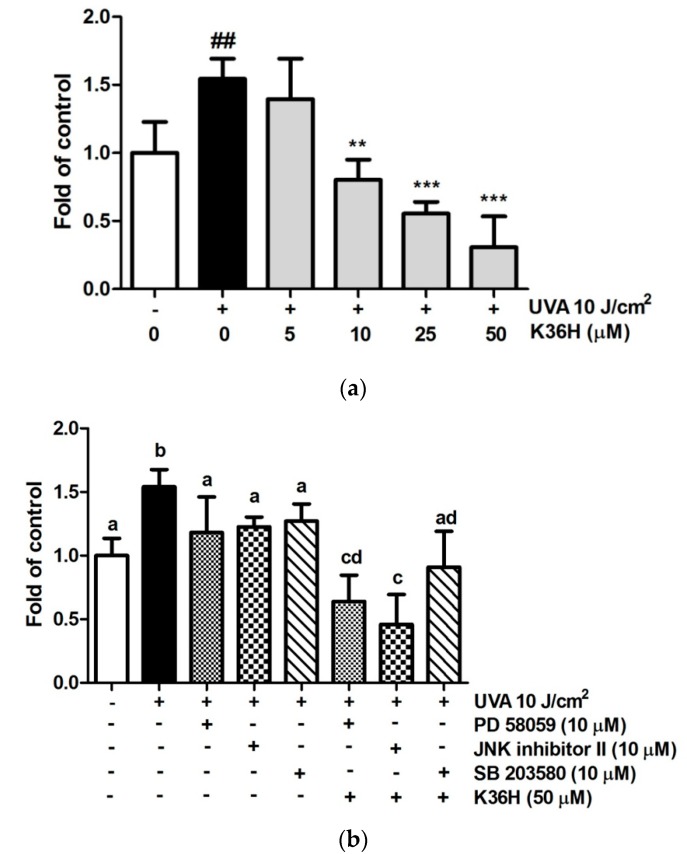
(**a**) Effect of K36H on NO level in HaCaT cells. Significant difference versus the nonirradiated group: ## *p* < 0.01. Significant difference versus the nontreatment group: ** *p* < 0.01; *** *p* < 0.001. (**b**) Effect of MAP kinase inhibitors and K36H on UVA-induced NO production in HaCaT cells. a-d: Groups sharing the same letter are not significantly different (*p* > 0.05) as revealed by LSD *post hoc* tests.

Excessive NO production occurs in the tissue injury of inflammatory diseases. Thus, we determined the contents of downstream inflammatory cytokines, such as NO and PGE_2_, using an ELISA kit. As shown in Figure 10a, UVA increased cellular NO production to 1.7-fold of the control group levels, whereas K36H significantly reduced NO content at doses higher than 10 μM. After K36H treatment, the NO level was reduced to 1.4, 0.8, 0.6 and 0.3 times the control levels. As shown in Figure 10b, UVA increase the NO concentration in keratinocytes. However, when treated with PD98059, JNK inhibitor II and SB203580 reduced the NO concentration. Cotreatment with K36H along with PD98059 and JNK inhibitor II further reduced the NO concentration. The results indicate that K36H inhibited NO concentration after UVA light exposure through the regulation of JNK and ERK pathways.

#### 3.7.3. Reduction of UVA-Induced PGE_2_ Production with K36H Treatment

**Figure 11:**
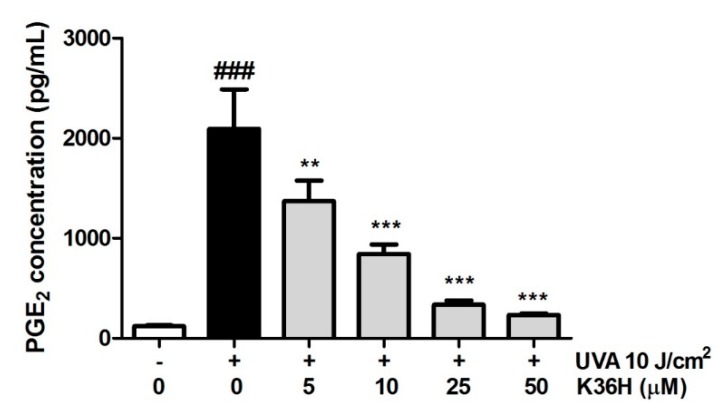
Effect of K36H on prostaglandin E2 (PGE_2_) production in human epidermal keratinocytes. Significant difference versus the nonirradiated group: ### *p* < 0.001. Significant difference versus the nontreatment group: ** *p* < 0.01; *** *p* < 0.001.

The PGE_2_ level of the cell culture medium increased from 123.0 ± 10.2 pg/mL to 2040.2 ± 409.7 pg/mL after keratinocytes were UVA irradiated (Figure 11). K36H treatment could dose-dependently reduce PGE_2_ production to 233.9 ± 15.4 pg/mL with 50-μM K36H treatment. UVA induced lipid peroxidation of the cell membrane to produce arachidonic acid, which produced Prostaglandins through COXs, amplifying the recruitment of inflammatory cells to the area [3,49].

In addition to antioxidative activity, K36H exhibited anti-inflammatory activity. Propolis exhibited downregulation of IL-12 and IL-6 expression and markedly ameliorated immune suppression triggered by UV irradiation [37]. Caffeamide derivatives inhibit UVB-induced inflammation-related proteins including COX-2, NF-κB and IL-6 expression in Hs68 and BALB/c hairless mice, indicating anti-inflammatory properties [50,51]. The results of the present study indicate that K36H efficiently reduces inflammatory mediators, including COX-2, iNOS and IL-6 proteins, as well as the concentrations of downstream inflammatory cytokines, such as NO and PGE_2_, indicating that K36H protects the skin from UVA-induced inflammation through the regulation of MAP kinases.

## 4. Conclusions

**Figure 12:**
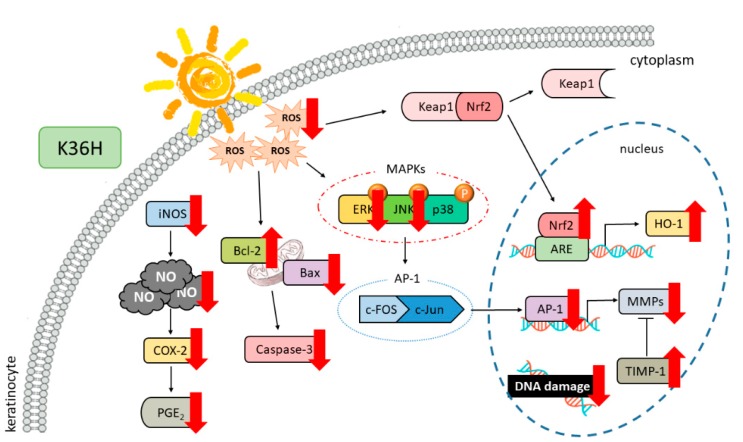
Effects of K36H on UVA-induced photoaging.

This study showed that K36H treatment prevented UVA-induced oxidative damage, such as DNA damage and apoptosis, in keratinocytes (Figure 12). K36H upregulated the self-oxidative defense system including Nrf2 and HO-1 to ameliorate UVA-induced damage. K36H had excellent free-radical-scavenging and anti-inflammatory properties. The photoprotective property of K36H may be due to its antioxidant activity.
